# Automatic Adaptive Algorithm for Delineation of Cerebral-Spinal Fluid Regions for Non-contrast Magnetic Resonance Imaging Volumetry and Cisternography in Mice

**DOI:** 10.21769/BioProtoc.5148

**Published:** 2025-01-05

**Authors:** Ryszard S. Gomolka

**Affiliations:** Center for Translational Neuromedicine, University of Copenhagen, Copenhagen, Denmark

**Keywords:** MRI, 3D-CISS, CSF space volumetry, Automatic 3D image segmentation

## Abstract

Magnetic resonance imaging (MRI) is an invaluable method of choice for anatomical and functional in vivo imaging of the brain. Still, accurate delineation of the brain structures remains a crucial task of MR image evaluation. This study presents a novel analytical algorithm developed in MATLAB for the automatic segmentation of cerebrospinal fluid (CSF) spaces in preclinical non-contrast MR images of the mouse brain. The algorithm employs adaptive thresholding and region growing to accurately and repeatably delineate CSF space regions in 3D constructive interference steady-state (3D-CISS) images acquired using a 9.4 Tesla MR system and a cryogenically cooled transmit/receive resonator. Key steps include computing a bounding box enclosing the brain parenchyma in three dimensions, applying an adaptive intensity threshold, and refining CSF regions independently in sagittal, axial, and coronal planes. In its original application, the algorithm provided objective and repeatable delineation of CSF regions in 3D-CISS images of sub-optimal signal-to-noise ratio, acquired with (33 μm)^3^ isometric voxel dimensions. It allowed revealing subtle differences in CSF volumes between aquaporin-4-null and wild-type littermate mice, showing robustness and reliability. Despite the increasing use of artificial neural networks in image analysis, this analytical approach provides robustness, especially when the dataset is insufficiently small and limited for training the network. By adjusting parameters, the algorithm is flexible for application in segmenting other types of anatomical structures or other types of 3D images. This automated method significantly reduces the time and effort compared to manual segmentation and offers higher repeatability, making it a valuable tool for preclinical and potentially clinical MRI applications.

Key features

• This protocol presents a fully automatic adaptive algorithm for the delineation of CSF space regions in 3D-CISS in vivo images of the mouse brain.

• The algorithm represents an analytical method for adaptive CSF regions separation based on cumulative distribution of brain image intensities and contrast calculation-based slice-wise region growing.

• Users can interactively alter the input parameters to modify the algorithm’s output in a variety of 3D brain MR and μCT or CT images.

• The algorithm is implemented in MATLAB 2021a and is compatible with all versions up to 2024a.

## Graphical overview


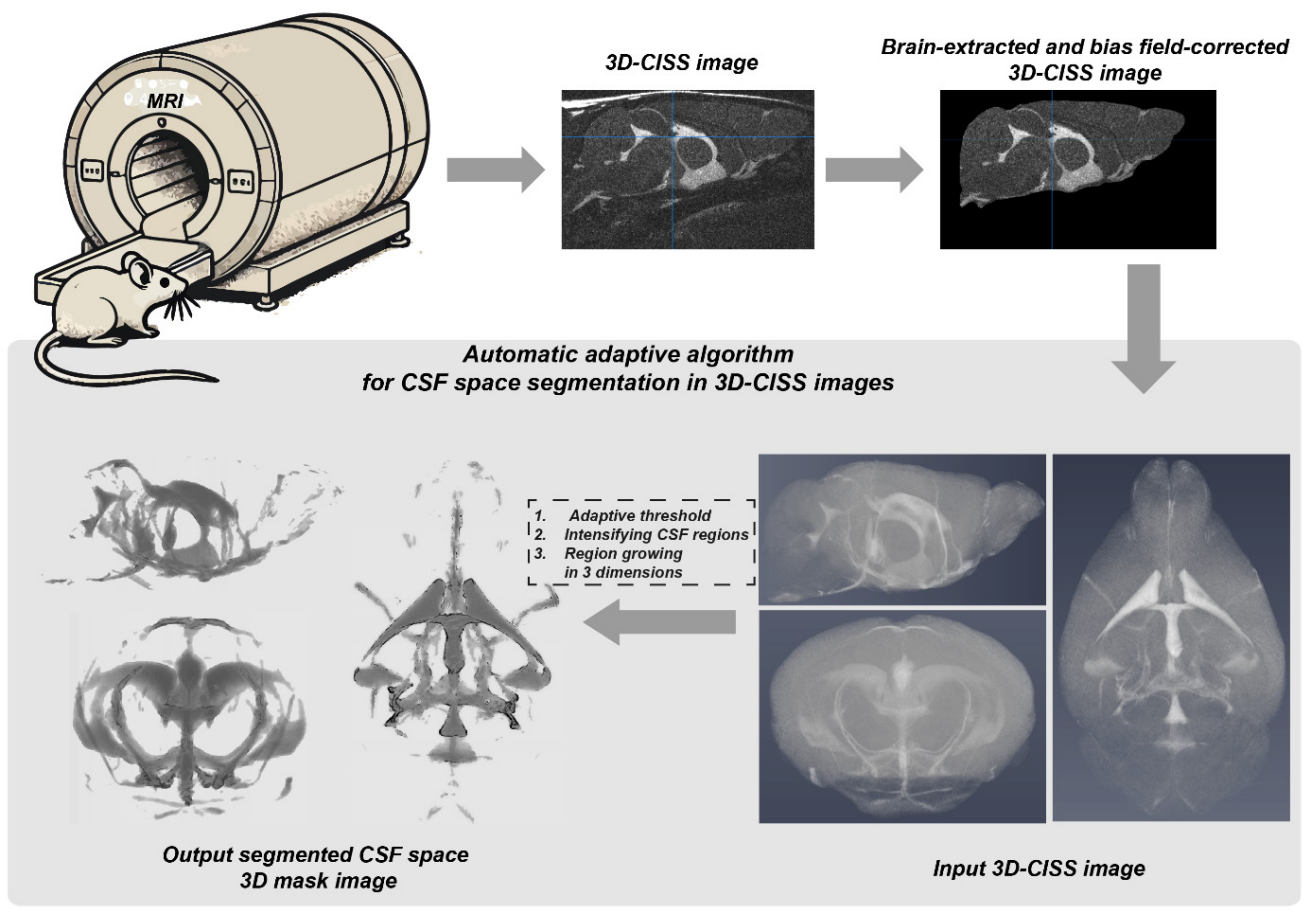


**From acquisition to automatically segmented CSF space image: a graphical abstract for application of the presented automatic adaptive algorithm for objective cerebrospinal fluid (CSF) space segmentation in 3D constructive interference steady-state (3D-CISS) images of the mouse brain.**

## Background

Magnetic resonance imaging (MRI) is an invaluable technique for anatomical and functional whole-brain in vivo imaging [1–3]. For non-invasive whole-brain MRI of the brain cerebrospinal fluid (CSF) space, a state-of-the-art T2-weighted spin-echo (SE) imaging can be used with inversion-recovery or saturation-recovery preparation pulses [4]. This provides high accuracy for the CSF space differentiation by means of “pure” image contrast [5], however, it requires remarkably long acquisition times reducing applicability, especially for 3D whole-brain imaging. An alternative considers rapid gradient-echo imaging based on steady-state techniques [6,7], encompassing radiofrequency-spoiled [8] gradient-echo (GRE) [9], to visualize high-intensity CSF regions. While providing shorter acquisition times than SE, GRE is strongly vulnerable to field inhomogeneities [10,11] requiring transmit field corrections [12]. Alternatively, strong T2 weighted contrast imaging with a true balanced steady-state free precession (TrueFISP) [13,14] allows higher signal-to-noise ratio (SNR) efficiency resulting in high spatial 3D resolution. TrueFISP is characterized with the image contrast close to that of SE [15], while for the time-to-repetition (TR) to time-to-echo (TE) ratio of 2:1 provides high T2 contrast [16–18]. It is easily affected by phase shift errors (banding artifacts) as it acquires constant phases across voxels [16,19,20]. A solution for CSF space volumetry uses maximum intensity projection of at least two combined TrueFISP acquisitions known as a 3D constructive interference steady-state sequence (3D-CISS), employed to visualize the structures at the skull base [21]. 3D-CISS provides strong T2-weighted CSF contrast and compensation for banding artifacts [22] and, due to balanced gradients, is almost invulnerable to flow artifacts (see Figure S1 for the mouse brain volume and CSF space visualization using 3D-CISS). This results in homogenous intensity distribution and high SNR and CSF space contrast [16–18,22,23]. Nevertheless, accurate delineation of the CSF space (similar to the segmentation of other structures in complex brain anatomy) is a critical part of MR image processing and analysis [24,25], especially when no external contrast agents are used for CSF space imaging. An accurate and reliable automatic approach is highly desired and possesses a clear advantage over the manual approach due to the time and effort required and higher repeatability. In the last decade, many segmentation methods for MR brain images in clinical [26–28] and preclinical [29–33] settings have been proposed, with a clear trend toward the application of artificial neuronal networks. However, in the vast majority of limited data available on-site, the analytical approach is fully sufficient. Recently, we have employed 3D-CISS along with a dedicated automatic adaptive algorithm in MATLAB for the objective delineation of CSF in aquaporin-4-null and wild-type mice [34]. Herein, we describe in detail this in-house developed algorithm, which provides automatic delineation of CSF space regions. The algorithm is based on the original method to study the cumulative distribution of intensities and contrast of the brain stroke regions in non-contrast computed tomography, characterized by similar brain tissue contrast and image SNR as in CISS [35,36], and provides objective delineation of the CSF space. The method is not limited to the purpose, and with input parameter adjustment, it can be used for segmentation of structures occupying different parts of voxel intensity distribution in the images.

## Equipment

**3D-CISS image acquisition**

1. 9.4 Tesla animal scanner (Bruker BioSpin, Ettlingen, Germany, model: BioSpec 94/30 USR) or another preclinical scanner

2. 240 mT/m gradient coil (Bruker BioSpin, model: BGA-12S) or other

3. Cryogenically cooled transmit/receive (Tx/Rx) quadrature-resonator (Bruker BioSpin, model: CryoProbe) or other preferably volumetric Tx/Rx resonator providing sufficient sensitivity

**Automatic CSF space delineation**

1. Standard PC with > 2 cores processor and > 8 GB RAM memory (herein, Intel Core i7-10700U, 32 GB RAM)

## Software and datasets

1. ITK-SNAP v3.x or later (www.itksnap.org, accessed July 2024) [37]

2. Analysis of Functional NeuroImages (AFNI; https://afni.nimh.nih.gov, accessed July 2024) [38]

3. FMRIB Software Library (FSL) v5.0 or later (https://fsl.fmrib.ox.ac.uk, accessed July 2024) [39]

4. MATLAB R2019a or later (https://www.mathworks.com/products/matlab.html, accessed July 2024). Current implementation developed in MATLAB R2021a, and tested in versions R2022b, R2023b, and R2024a.

5. All data code is available via GitHub (https://github.com/RSG1UCPH/CSF-space-segmentation-in-3D-CISS.git)

## Procedure

**A. Magnetic resonance imaging**

The described algorithm was originally employed for objective assessment of the volumes and structural differences between the brain CSF spaces in aquaporin-4-null and wild-type littermate mice, based on 3D-CISS images [34]. MR imaging was performed at 9.4 Tesla (BioSpec 94/30 USR, Bruker BioSpin, Ettlingen, Germany) equipped with 240 mT/m gradient coil (BGA-12S, Bruker BioSpin) and cryogenically cooled transmit/receive (Tx/Rx) quadrature-resonator (CryoProbe, Bruker BioSpin). The imaging protocol consisted of T2-weighted 2D rapid acquisition with relaxation enhancement (RARE) for reference spatial planning and two 3D-TrueFISP acquisitions with orthogonal phase encoding directions for CSF space imaging. For details of the imaging protocol, please refer to the original paper [34].

**B. MATLAB and toolboxes**

MATLAB can be installed from the MathWorks product webpage and is available for Windows, Linux, and Mac operating systems (OS). For our purposes, a Windows OS was used. Additional information regarding available MATLAB products and installation on different OS can be found on the producer’s web page (www.mathworks.com/products/matlab.html, accessed July 2024).

The following MATLAB toolboxes are required to run the algorithm:

A. Image Processing Toolbox (functions: *niftiread, bwboundaries, medfilt3, permute*, and *imshow*)

B. Statistics and Machine Learning Toolbox (function: *prctile*)

**C. Step-by-step protocol description**

*Note: Skip steps C1–3 if a 3D-CISS image is already available.*

1. Image acquisition [34]: Acquire two 3D-TrueFISP volumes, each with opposite phase encoding direction (i.e., 0° and 180°).

2. Save acquired images in NIfTI format.

3. 3D-CISS image formation:

a. Perform motion-correction of 3D-TrueFISP images (if more than one repetition was used per each acquisition) 10 times or until no further improvement [*3Dvolreg()* function in *AFNI*], aiming to reduce the influence of random motion on the subsequently computed 3D-CISS image.

b. Calculate averaged 3D-TrueFISP image from all repetitions per acquisition using opposite phase encoding direction.

c. Realign the second averaged (and subsequent if available) 3D-TrueFISP volume (i.e., acquired with 180° phase encoding direction) to the first volume (i.e., acquired with 0° phase encoding direction) using rigid-body registration [6 d.f.; *3Dvolreg()* function in *AFNI*] for subsequent calculation of 3D-CISS volume.

d. Calculate 3D-CISS image as a maximum intensity projection from two co-registered 3D-TrueFISP volumes, allowing to obtain an almost banding artifacts-free image (see example of acquired 3D-CISS image in Figure S2A).

4. Perform semi-automatic segmentation of brain parenchyma (Figure S2B) in calculated 3D-CISS image using the *Segment 3D* tool in ITK-SNAP or other semi-automatic or automatic method or software available for this purpose. Brain parenchyma is considered here as the brain tissue volume surrounded by dark regions of the skull image and including intracerebral vessels, as defined in [34] (see example in Figure S2C–E). This step is necessary to reduce the computational burden on subsequent CSF space segmentation in MATLAB, by removing the regions outside the brain parenchyma image from further analysis.

5. To correct for intensity inhomogeneities coming from the B0 field and the profile of the Tx/Rx coil (in original method surface profile of the CryoProbe [34]), perform N4 bias field correction in the brain-extracted parenchyma using FAST tool in FSL (0.5 sigma, 20 mm FWHM, 4 iterations).

6. Finally, perform automatic CSF space segmentation using the dedicated adaptive algorithm described below in MATLAB. For a single, bias-corrected, and brain-extracted 3D-CISS volume, the algorithm separates the ventricular and perivascular CSF spaces from the brain parenchyma image in three dimensions, and by means of four consecutive steps based on the following functions:

• ***CSF_volumetry()***: The main function that runs the entire processing pipeline.

• ***mask_brain_only()***: Isolates the brain regions from the image.

• ***intensify_CSF()***: Enhances the CSF space image seed regions based on an adaptive thresholding.

• ***grow_regions()***: Refines the seed regions by comparing their intensity and contrast to that of surrounding brain parenchyma, in sagittal plane.

• ***grow_regions2()***: Further refines the fine-tuned seed regions from the previous step, in axial and coronal planes.

• ***SNRstats()***: Computes basic statistical properties of the input image.

• ***save_nifti()***: Function for saving the results of the automatic segmentation algorithm.

**Figure 1.**
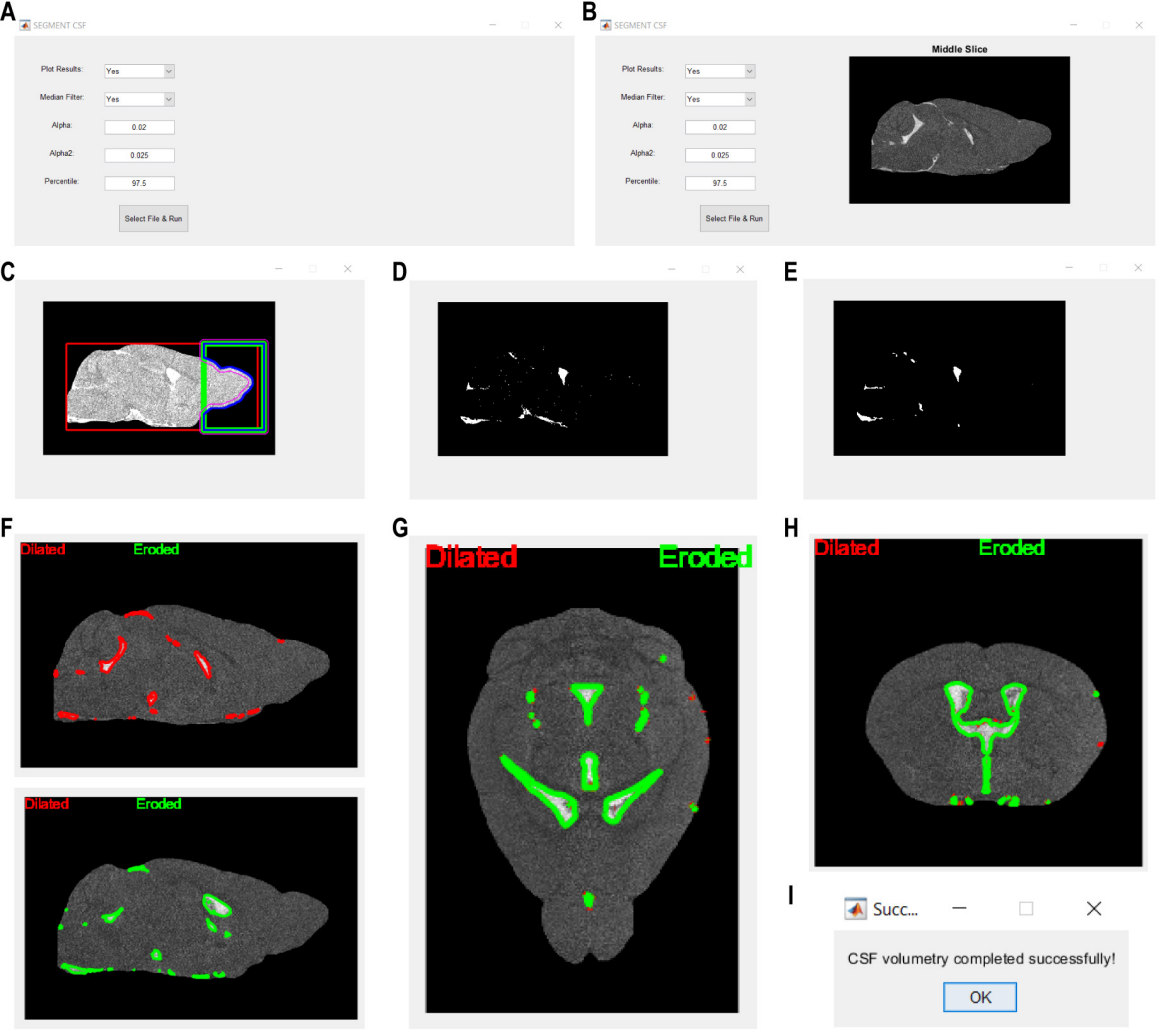
Action steps of the presented automatic adaptive algorithm for delineation of cerebrospinal fluid (CSF) space in non-contrast 3D constructive interference steady-state (3D-CISS) images of the mouse brain. (A) User interface for the CSF segmentation algorithm, where parameters such as plotting results, applying median filtration, and adjusting α and α_2_ (see step 4b) and percentile values (see steps 2–4) can be done before selecting a file and running the processing. **(B)** After the image is selected, its mid-slice is displayed in the window on the right side from the user interface input text boxes. **(C)** Brain parenchyma is enclosed within a calculated bounding box for the removal of non-continuous and not adjacent to the parenchyma image high-intensity regions (see step 1). Parenchymal regions are highlighted in each analyzed slice, with different outlines indicating areas of interest, namely: red outline, brain parenchyma bounding box (*BOX*, see step 1); green outline, bounding box covering only the olfactory and optic nerves area (distal 25% length of the red box + arbitrary, set to 16 voxels, extension outside the red box to cover any regions potentially removed by the red box); pink outline, refined brain parenchyma boundaries within the green box outline; blue outline, final brain parenchyma outline after removal of high-intensity parenchyma image regions not adjacent to the brain (*BOX2*, see section 1). **(D)** Binary mask image showing the segmented CSF seed regions before and **(E)** after optional 3D median filtration (see steps 2 and 3). The CSF seed regions mask further undergoes region growing in **(F)** sagittal, **(G)** axial, and **(H)** coronal planes, considering regions dilation (red outline) followed by erosion (green line; see step 4). **(I)** Confirmation dialog indicates successful completion of the CSF space segmentation, signaling the end of the automated analysis.

*Note: The algorithm is available online (* *https://github.com/RSG1UCPH/CSF-space-segmentation-in-3D-CISS.git* *) and is equipped with a simplified user interface (see Figure 1A and B, function CSF_volumetry_gui()), allowing modification of the most important input parameters and analyzing a single image at a time. The CSF_volumetry() function can be used without the user interface and can be adapted for analyzing multiple files consecutively in a loop. For supplementary instructions on the usage of the functions, please refer to the README.txt file available along with the original code in MATLAB.*

**D. CSF space segmentation in an input 3D-CISS image**

The CSF space segmentation in an input 3D-CISS image takes place in the following steps:

1. Computation of the bounding box enclosing the brain and removal of high-intensity regions not adjacent to the brain parenchyma as branches of the optic nerve’s residual after the semi-automatic brain image extraction.

The bounding box is automatically computed based on both minimization and maximization of the voxel intensity variance slice-wise, separately in three orthogonal planes. The parenchyma volume surrounded by the bounding box is being enclosed, and the solitary regions are removed based on their geometrical properties calculated slice-wise in the sagittal plane: *eccentricity ≥ 0.5, roundness ≥ 0.5, perimeter < 0.005% of the brain parenchyma voxels count*. The resulting brain image mask is geometrically dilated with a disk kernel of 11 pixels in diameter (Figure 1C) to enclose potentially removed or non-continuous parenchymal regions. The considered non-continuity appears in the case of residuals from banding artifacts at the borders of the skull and the ethmoidal bone. Subsequently, brain parenchyma image volume is updated according to the resulting mask for further automatic segmentation of the CSF space.

**Important:** This method assumes that the brain-extracted and bias-corrected brain parenchyma image is realigned to orthogonal axes and placed in the center of the 3D-CISS image (see example file: *Test_brain1.nii.gz and Test_brain2.nii.gz*).

***Function Definition and Description***

**The function *[BOX] =* m*ask_brain_only(image, plt)*** isolates the brain region in a 3D volume by creating a binary mask that excludes non-brain areas.

**Inputs:** *image* (3D image volume) and *plt* (flag for plotting intermediate results).

**Output:** *BOX* (binary 3D mask for the brain image).

a. Middle slice calculation

The middle slice in the sagittal plane is found by averaging two central slices of the 3D-CISS volume. This helps to identify the central region of the brain and to subsequently calculate the extents of the bounding box. The mid-slice in the coronal plane is calculated after spatial coordinates of the bounding box are calculated in sagittal and axial planes.

b. Bounding box calculation

For calculation of the bounding box enclosing the brain parenchyma image in three dimensions, a variance is calculated across columns and rows of the mid-sagittal slice to identify brain parenchyma extents (with non-zero variance). Based on minimization and maximization of the calculated non-zero variance in sagittal, axial, and coronal planes, coordinates defining the bounding box are determined.

c. Binary brain mask

Two binary masks (*BOX* and *BOX2*) are initialized. *BOX* is the main mask, and *BOX2* is a secondary mask to help refine the 3D brain-encapsulated volume. For each slice, the masks are refined by:

i. Removing areas with significant intensity.

ii. Dilating the mask to fill any gaps and smooth boundaries.

iii. Selecting regions based on shape properties (circularity, eccentricity, perimeter).

iv. Eroding the mask and filling holes.

The main mask (*BOX*) and the refined secondary mask (*BOX2*) are combined to produce the final brain region mask.

d. Applying the bounding box

The refined mask of the bounding box enclosing the brain parenchyma image in three dimensions is applied to the original *image*, and the result is saved into *new_image* 3D volume.

e. Plotting

If plotting is enabled, intermediate results are displayed to visualize the mask refinement process.

*Note: The bounding box calculation is an integral part of the described method, and its omission is generally not recommended. However, users who thoroughly understand the method and code in MATLAB and are confident that the brain parenchyma is well separated in their input image may choose to bypass this step by commenting relevant fragment of the original code. Due to this possibility, the updated with the bounding box new_image, originally defined in the MATLAB code, is defined in the following functions as the image.*

**Important:** The bounding box calculation assumes that, in 2D slice of the brain image volume, the olfactory bulb region is placed toward the end of the image, considering [0,0] point as the left-upper origin of the slice (see example file: *Test_brain1.nii.gz and Test_brain2.nii.gz*).

2. Calculate image SNR-related adaptive threshold parameter *xp* based on the mean and standarddeviation of the 3D-CISS image voxel intensity distribution.

*Note: This step takes place inside the main CSF_volumetry() function.*

a. Flattening the input image

After removing the obsolete regions using the calculated bounding box, the brain-extracted 3D-CISS input image is flattened into a vector *vect*, and only non-zero image intensities are considered. This is performed in case any preprocessing step introduces zero or negative values or if other types than MR image are used for analysis (i.e., computed-tomography image that possesses voxels of < 0 in Hounsfield units).

b. Calculation of non-zero intensity distribution

The length, mean, and standard deviation of the vector *vect* are calculated and saved into matrix *V* for recording in case of multiple image analysis. A parameter *xp* is calculated based on the mean (*μ_vect_*) and standard deviation (*σ_vect_*) of the non-zero intensities from *vect*:

$$xp=\frac{\sigma_{vect}}{{µ}_{vect}+\sigma_{vect}}$$

The parameter *xp* provides an adaptive correction for the subsequent depiction of CSF space seed regions, reflecting the properties of the distribution of intensities in the brain-extracted 3D-CISS image, i.e., a surrogate for the assessed image SNR.

c. Adjust the predefined percentile threshold of the brain parenchyma intensities distribution in the input image

The initial percentile value *P* for separating the CSF space from the brain parenchyma image is equal to 95.5, assuming that the CSF space intensities in the brain-extracted 3D-CISS brain parenchyma image are above the 95^th^ percentile of the image’s cumulative distribution of non-zero voxel intensities. Based on the SNR surrogate *xp*, the adjustment of the percentile *P* considers three conditions:

i. For images of SNR > 4 (i.e., low influence of Rician noise; CSF space is well-defined in 3D-CISS image, so the percentile *P* threshold is shifted to higher values: *P = 95.5 + xp*).

ii. For images of 4 ≥ SNR > 2 (i.e., existing influence of Rician noise; differentiation of the CSF space is affected by noise, but the input image has great T2 contrast, so the separation threshold is unchanged: *P = 95.5*).

iii. For images of SNR ≤ 2 (i.e., strong influence of Rician noise; differentiation of CSF space is more difficult due to considerable contribution of noise. Differentiation of seed regions requires including a larger part of the voxel intensity distribution, so the percentile *P* threshold is shifted to lower values: *P = 95.5 - xp*).

*Note: The correction factor xp accounts for subtle intensity changes and does not result in the threshold going below 92^nd^ and exceeding the 97^th^ percentile of the brain-extracted and bias-corrected 3D-CISS image voxel intensity distribution.*

3. Perform initial CSF space segmentation by means of an adaptive intensity threshold and calculation of the cumulative distribution of voxel intensities > 0 from the brain-extracted and bias-corrected 3D-CISS volume.

***Function Definition and Description***

**The function *[mask] = intensify_CSF(image, P)*** processes the brain-extracted and bias-corrected 3D-CISS image to adaptively enhance (CSF) space regions and to depict the CSF space seed regions for subsequent automatic region growing segmentation. The function uses intensity distribution normalization and *P* threshold parameter from the prior step.

**Inputs:** *image* (3D image data) and *P* (percentile value used for adaptive thresholding of the image intensities).

**Output:** *mask* (binary 3D mask image highlighting the CSF seed regions for further region growing algorithm).

a. Flattening the input image:

The brain-extracted and bias-corrected 3D-CISS input image is flattened into the vector *vect*, and the mean and standard deviation of non-zero voxel intensity distribution is calculated as in step 2a (see above). This calculation is repeated in case the user wishes to use the *intensify_CSF()* function independently.

b. Image intensity distribution normalization:

Each voxel of the input image is rescaled to enhance the distinction of the CSF space regions, assuming that CSF intensities reflect those > 90^th^ percentile of the aggregated non-zero intensities distribution within the *image*. The image is normalized by subtracting 1.33 times the standard deviation of non-zero intensities (*σ_vect_*) and then dividing by the *σ_vect_*. This rescaling factor is applied to ensure that all high-intensity CSF space regions are included in further consideration, along with their closely neighboring regions affected by the partial volume, but not the rest of the brain parenchyma image:

$$intensified\_CSF\_image=\frac{\left(image-1.33\times \;\sigma_{vect}\right)}{\sigma_{vect}}$$

The rescaled (normalized) voxel intensities are saved using a floating point precision in the range between − 1.33 × *σ_vect_* and the new distribution peak close to the image SNR defined as *µ_vect_/σ_vect_* (the maximum rescaled intensity ~10 and the mean value varying between 3 and 5 among all analyzed images in the original study [34]).

c. Adaptive thresholding:

All the rescaled voxels possessing negative intensity (i.e., belonging to the brain parenchyma image) are assigned to 0, and a new aggregated distribution of the rescaled voxels of > 0 intensity is computed. Subsequently, the image intensity at the *P* percentile (see step 2c) of the new non-zero voxel intensity distribution is calculated and denoted as *Px*. All voxels of intensities ≤ *Px* are assigned to 0 to keep only the high-intensity CSF seed regions (Figure 1D).

d. Binary CSF space mask conversion:

All remaining non-zero intensities are set to 1, creating a binary *mask* image where the CSF seed regions are highlighted.

*Note: An optional 3D median filtration is available after the intensify_CSF() function is executed (Figure 1E) and is recommended if the computation of mask image leads to the depiction of multiple non-continuous and spurious segments due to low SNR or high influence of Rician noise in the input image. However, it is also recommended to compare the results of segmentation with and without 3D median filtration.*

4. Final segmentation: Apply region growing algorithm to reconsider borders of the seed CSF space regions.

***Functions Definition and Description***

**The function [*mask2] = grow_regions(mask, image, plt, xp)*** reconsiders the borders of the CSF space seed regions in the sagittal plane, based on their intensity and contrast to the neighboring regions.

Similarly, subsequently called two consecutive times function **[*mask3] = grow_regions2(mask2, image, plt, xp, mfilt)*** reconsiders the CSF space regions in axial and coronal planes, respectively. The function updates the CSF space *mask* based on the output from the previously called function.

**Inputs:** *mask* (binary mask image with initial or updated seed CSF space regions), *xp* (percentile adjustment for the adaptive intensity thresholding), *image* (original brain-extracted and bias-corrected 3D image), *mfilt* (flag for optional slice-wise 2D median filtration), and *plt* (flag for plotting intermediate results).

**Output:** *mask* (binary 3D mask image with reconsidered CSF space image regions).

a. Flattening the input image

The brain-extracted and bias-corrected 3D-CISS input image is flattened into a vector *vect*, and the mean and standard deviation of non-zero voxel intensity distribution is calculated as in step 2a (see above). As before, this calculation is repeated in case the user wishes to use this function independently from the whole algorithm.

b. Region growing

The function processes each corresponding slice of the *image* and *mask* volumes parallelly, excluding the last border slice. For each slice of the *image*, its original and its zero-padded (extended by *n* zero-value voxels in both sides of two orthogonal directions, to account for the largest region growing kernel specified below) copies are made to avoid manipulations in the original image. If the analyzed *image* slice contains any positive values (i.e., CSF regions are present), boundaries of the seed CSF regions are computed in the corresponding slice of the *mask* volume (Figure 1F–H).

**Dilation:** To ensure that only voxels belonging to the CSF space and not affected by the partial volume from the surrounding parenchyma are considered, these are included in the final CSF space *mask* if their intensity values are ≥ (*97.5^th^ - xp)* percentile (i.e., *μ_d_* + 2 × *σ_d_*) of the aggregated voxel intensity distribution from the *image* and fulfill the contrast calculation-based condition for continuity of the CSF space. The boundary contrast is calculated as an absolute relative CSF to brain parenchyma contrast in the *image*, considering the mean of *n* voxels intensities at each side from the boundary voxel in horizontal/vertical/diagonal directions. The contrast is calculated for *n* from 1 to 4 in the sagittal and from 1 to 3 voxels in the axial and coronal planes. The voxels at *n* ^th^ distance from the boundary are included in the updated CSF *mask* if their absolute relative contrast is below the threshold α = 2%.

**Erosion:** Similarly, the updated CSF *mask* is recalculated, and the voxels are reconsidered for exclusion using the same method. Herein, the contrasts are calculated for a smaller *n* from 1 to 2 in the sagittal and from 1 to 3 in the axial and coronal planes, to avoid removing small regions belonging to the perivascular space around the main cerebral arteries. The voxels at *n* ^th^ distance are removed from the updated CSF mask if their intensity in the original CISS image is ≤ (*95.5^th^ - xp)* percentile [for sagittal, and < (*95.5^th^ - xp)* for the axial and coronal planes] of the aggregated intensity distribution, and the absolute relative contrast to the respective boundary voxel is above α_2_ = 2.5% (for sagittal, or above and equal to α_2_ for the remaining planes).

The refined slices are added to the final output mask.

*Note: The version of the algorithm equipped with the user interface has additional input structure params allowing for changing the 97.5^th^ percentile threshold as well as the α and α_2_ parameters so that the user can define the conditions for the region growing algorithm.*

c. Optional morphological filtering

If median filtering *mfilt* is enabled, a 2D median filtration with a 3 × 3 voxels kernel is applied to every slice of the updated CSF space mask image, to remove remaining false-positively segmented single voxels and to enclose wrongly opened larger regions (***grow_regions2() function only***).

d. Plotting

If plotting *plt* is enabled, intermediate results are displayed to visualize the CSF space mask refinement process (Figure 1F–H).

5. Saving of the output

The final median-filtered and unfiltered output CSF space mask volumes are saved in NIfTI format into the directory of the original input file. Names of the output files reflect the original input file name appended with “CSF_mask_medfilt_final.nii” and “CSF_mask_final.nii” in case of median-filtered and unfiltered 3D volumes, respectively. After the automatic algorithm successfully finished processing, a confirmation dialog opens in a separate window (Figure 1I).

*Note: Execution time for the algorithm depends on the number of processor cores, available memory, image resolution, and the size and number of segmented ROIs. For high-resolution 3D-CISS whole-brain images with a voxel size of (33 μm)^3^ (as analyzed in [34], image sample provided in the Supplementary information), the processing time in MATLAB ranged from 3.5 to 4.5 minutes using a personal computer equipped with an Intel Core i7-10700U 6-core processor and 32 GB of RAM.*

## Data analysis

The presented algorithm was intentionally developed for automatic CSF space delineation in high-resolution 3D-CISS images. However, we encourage applying the algorithm for the analysis of other types of MRI or micro-computed tomography images, by interactively changing the input parameters to depict different parts of the image voxel intensity distribution (see user interface in Figure 1A and B). Usage of the algorithm does not require substantial skills in MATLAB; however, any modifications require an understanding of the MATLAB code, organization of the 3D image data matrix, and mathematical reasoning behind the algorithm.

## Validation of protocol

The capabilities of the algorithm were tested in 3D-CISS images acquired with (50 μm)^3^ and (33 μm)^3^ isometric voxel size, presented during the ESMRMB 2021 conference [40] in the original research paper [34].

## General notes and troubleshooting

**Usage of 3D-CISS for CSF space imaging**

When acquiring 3D-CISS images, it is important to separately consider the acquisition of 3D-TrueFISP volumes, which are significantly affected by banding artifacts. Despite compensatory measures such as increasing resolution, bandwidth per voxel, and reducing both time-to-repetition and time-to-echo, these artifacts remain challenging to eliminate in clinical (low-field) and especially preclinical (high-field) MR systems. They are particularly prominent at the skull base and other regions where magnetic field susceptibility distortions are unavoidable, resulting in an overall loss of image quality. To better address these issues, we recommend acquiring 3D-CISS using four (instead of two) TrueFISP acquisitions, each in orthogonal phase encoding directions (0°, 180°, 90°, and 270°). It is also important to note that our study's evaluation may be affected by the parabolic CryoProbe sensitivity profile. To mitigate this, we recommend performing a bias field correction of 3D-CISS images as we did in the original study [34,40].

**Delineation of CSF space in 3D-CISS images using the proposed method**

We have demonstrated that our automatic CSF segmentation in 3D-CISS images is effective in depicting subtle differences in CSF volume between wild type and animals lacking the aquaporin-4 channel [34]. With the highest possible spatial resolution achieved using our setup and only two phase-encoding directions applied for CISS image calculation, our results showed good differentiation of the CSF space without the need for contrast agent injection. It is worth noting that using four encoding directions in our setup would increase acquisition time to up to three hours and might introduce additional biases from variability in anesthesia levels or due to prolonged animal restraint. Additionally, single voxel layers, fuzzy or smaller than 33 μm in any dimension regions, might have been missed due to partial volume effects or residuals from banding artifacts. Therefore, besides acquiring TrueFISP in more encoding directions, we also recommend applying more averages (repetitions) for CISS image formation if the protocol time allows. Finally, we encourage researchers to use this algorithm and further validate its action in their images, especially in the presence of a reliable and known segmentation ground truth.

## Supplementary information

The following supporting information can be downloaded here:

The algorithm implementation in MATLAB can be downloaded in https://github.com/RSG1UCPH/CSF-space-segmentation-in-3D-CISS.git). Additionally, exemplary 3D-CISS volumes can be found here:

1. “Test_brain1.nii.gz” – high-resolution 3D-CISS volume acquired using (33 μm)^3^ using voxels volume

2. “Test_brain2.nii.gz” – 3D-CISS volume acquired using (100 μm)^3^ using voxels volume

3. Figure S1. 3D-CISS volume

4. Figure S2. 3D-CISS image preprocessing steps
